# Validation of the Comprehensive Feeding Practices Questionnaire with parents of 10-to-12-year-olds

**DOI:** 10.1186/1471-2288-11-113

**Published:** 2011-08-09

**Authors:** Elisabeth L Melbye, Torvald Øgaard, Nina C Øverby

**Affiliations:** 1 University of Stavanger, Norwegian School of Hotel Management, 4036 Stavanger, Norway; 2 University of Agder, Department of Public Health, Sport and Nutrition, PO Box 422, 4604 Kristiansand, Norway

## Abstract

**Background:**

There is a lack of validated instruments for quantifying feeding behavior among parents of older children and adolescents. The Comprehensive Feeding Practices Questionnaire (CFPQ) is a self-report measure to assess multiple parental feeding practices. The CFPQ is originally designed for use with parents of children ranging in age from about 2 to 8 years. It is previously validated with American and French parents of children within this age range. The aim of the present study was to adapt and test the validity of this measure with parents of older children (10-to-12-year-olds) in a Norwegian setting.

**Methods:**

A sample of 963 parents of 10-to-12-year-olds completed a Norwegian, slightly adapted version of the CFPQ. Scale analyses were performed to test the validity of the instrument in our sample.

**Results:**

Although a few problematic items and scales were revealed, scale analyses showed that the psychometric properties of the slightly adapted, Norwegian version of the CFPQ were surprisingly similar to those of the original CFPQ.

**Conclusions:**

Our results indicated that the CFPQ, with some small modifications, is a valid tool for measuring multiple parental feeding practices with parents of 10-to12-year-olds.

## Background

Much of our eating behaviors are formed in early childhood and most behaviors are modeled after important caregivers of the child, primarily the parents [1]. Furthermore, parents shape children's early experiences with food and eating [2], and can affect children's diet and eating behaviors in numerous ways. For instance: by encouraging them to eat certain foods, by restricting certain foods, or by passively allowing certain foods in the regular diet. Other important parent-related determinants of children's eating behaviors are the physical and emotional environment in which eating behaviors are developed [3]. Hart, Bishop, and Truby [4], have stated a need for increased knowledge about parental influence on children's eating behavior. Also Zeinstra [5] has suggested that further research on child eating behavior should focus on the role of parental strategies in shaping children's food preferences and consumption.

A barrier to this literature has been a lack of validated instruments for quantifying parental feeding behaviors and styles [6]. Thus, comparability of studies has been a challenge. In a review of 22 studies [7], only the Child Feeding Questionnaire (CFQ) [8] was cross-validated in different parental samples and used in multiple settings. Furthermore, most previous measures of parental feeding practices have included just a few feeding practices, such as restrictive feeding and pressure to eat. These practices are aspects of control over child food intake, and are typically measured with the CFQ [8]. Although controlling feeding practices seem to be widely used by parents in an attempt to secure a well-balanced diet for their children [7], some studies have proved counterproductive effects of these strategies, as parents who exert too much control over child food intake tend to have children with an increased preference for high-fat foods and higher levels of snack-food intake [9].

The emphasis on parental control in previous feeding practices measures may have left other important feeding practices rather underexplored. This is especially true for feeding practices that are associated with desirable outcomes in children [10]. Parental modelling of healthy eating and exposure to healthy foods are examples of feeding practices that may be effective [11-14]. The extent to which parents try to teach their children about nutrition is another aspect not examined in the previous measures of parental feeding practices [10]. However, more recent research has suggested that additional feeding practices such as these can also be measured in parents and might impact child outcomes [15]. The Comprehensive Feeding Practices Questionnaire (CFPQ) [10] is an extension of previous feeding practices measures, and represents a more complete range of feeding practices that may be relevant to child outcomes. It consists of 49 items representing 12 dimensions (subscales), each including 3-8 items. Initial testing of the CFPQ with American parents of 2-to-8-year-olds showed reasonable validity and reliability [10]. An analysis of nine CFPQ subscales with French parents of 4-to-7-year-olds demonstrated reasonable validity and reliability in this sample as well [15].

Although the CFPQ appears to be a promising instrument for measuring multiple parental feeding practices, it's important to note that this instrument was designed to measure feeding practices in parents of young children (about 2 to 8 years of age). Zeinstra [5] suggests that children's cognitive development influences the strategies that parents use to shape the eating behavior of their children. Thus, feeding practices measures developed for parents of young children will not necessarily be valid for parents of older children and adolescents. As far as we know, only one previous study has validated a (pure) feeding practices measure with parents of older children and adolescents. In this study by Kaur et al [16], a modified version of Birch's CFQ was validated in a multi-ethnic sample of 260 parents of 10-to-19-year-olds (mean age:15 years). The psychometric properties of the modified CFQ were found to be similar to those of the original CFQ. However, consistent with the evolving independence of adolescents, the factor scores for the controlling feeding practices measured by the CFQ decreased with increasing age of the adolescent. That is; controlling feeding strategies seemed to be less used by parents of older children and adolescents than for younger ones. Nevertheless, the fact that parents are considered to be an important social agent impacting upon children's diets, also applies to older children and adolescents [17].

So far, most studies of parental influence on child eating behavior have focused on young children. In the present study, we have focused on feeding practices in parents of children on the onset of adolescence (10-12-year-olds). Adolescence is the period from about the age of eleven to the late teen years, and represents a transitional stage from childhood to adulthood. It is characterized by the elaboration of identity, and it is a time of growing independence when individuals want to make their own decisions including what and when to eat [18,19]. This stage is typically a time of gradual shift from parental to peer influence [20]. Thus, during adolescence parental influence over food choice may be displaced by the effects of advertising and peer pressure [21], and the age at which these changes set in appears to be diminishing [22]. However, the eagerness of adolescents to take over responsibility for food choice is not necessarily matched with their ability to make healthy food decisions. Adolescents have a reputation for unhealthy food choices [23,24], and interventions directed towards this group of the population have had mixed success [25]. Furthermore, research has found that adolescents understand at an abstract level the (un)healthiness of foods, but have limited concern about future health [26]. Therefore, the influence of parents should be assessed at all stages of this "hand-over-of-control" period to assist in the development of concurrent parental and peer group intervention programs [27]. The rationale for focusing on 10-to-12-year-olds in the present study is that children this age are still highly influenced by parents. Accordingly, it might be easier to implement intervention programs involving parents among individuals within this age range than among older ones.

Given the lack of validated instruments measuring feeding practices that might be relevant for parents of older children and adolescents, we aimed to test the validity of the CFPQ with Norwegian parents of 10-to-12-year-olds to check if it is a suitable tool for measuring feeding practices in this part of the population. We believe that development and validation of broad feeding practices measures such as the CFPQ is of great importance for applied research aiming to develop interventions to improve children's and adolescents' diets, whether it is for public health purposes or for clinical purposes.

## Methods

### Procedures and participants

For practical reasons, participants were recruited through primary schools in two neighbouring municipalities (Gjesdal and Sandnes) in the South-Western part of Norway. All primary schools in these municipalities were asked to participate in the study, and 18 out of 25 schools agreed to participation. Both urban and rural schools were included in the study to secure variance in our data. In total, 1466 parents of children aged 10 to 12 years (grade 5 and 6 students) were invited, forming a cluster sample. Survey packages including information letters, consent forms and self-administered questionnaires were distributed to the children at school with instructions to bring them home to be completed by one of their parents (the parent most involved in home food issues) within three days. Strategies to enhance the response rate included information about the aim and importance of the study, reassurance that respondent privacy would be protected, that participation would require little effort (not difficult or time consuming) and that participation involved a lottery with the possibility of winning a gourmet restaurant meal.

The study was approved by the Norwegian Social Sciences Data Services (NSD), which is the Privacy Ombudsman for all the Norwegian universities, university colleges and several hospitals and research institutes. The study protocol was also submitted for consideration and approval by the Regional Committee for Medical and Health Research Ethics (REK, Vest). However, the ethics committee decided that the Norwegian Act on Medical and Health Research (The Health Research Act) [28] did not apply to the present study, as the individual health information included in this project was considered marginal. Thus, the study could be conducted without their approval.

We received 963 completed questionnaires (66%). Response rates ranged from 44 to 93% among participating schools. Of the 963 respondents, 820 (85%) were mothers, 118 (12%) were fathers, and 11 (1%) were other caregivers (e.g. stepmother/stepfather). Fourteen participants (2%) did not report their relationship with the child. The average age of the participants were 39,8 years and 91% of the sample was of Norwegian or other Nordic origin (8% had their origin outside the Nordic countries, 1% did not report country of origin).

### Measures

The survey questionnaire included a Norwegian version of the CFPQ, items from three related attitude scales, and demographic questions.

#### CFPQ

The CFPQ items were translated from English into Norwegian by the first author (ELM) and a random sample of 10 items were back translated into English by the third author (NCØ). Both translators are experienced nutritionists, Norwegian native speakers and fluent speakers of the English language. A linguist assessed the quality of the translation by evaluating the semantic equivalence between the two English versions. The quality was considered very good as the meaning of the items were retained after translation/back translation.

The CFPQ was originally developed to measure multiple feeding practices among parents of children in the age span from about 2 to 8 years. In the present study the questionnaire was slightly adapted to fit parents of 10 to 12 year old children. The adaptation was guided by assessment/pre-testing of the instrument among Norwegian parents of 10-to-12-year-olds (4 mothers, 2 fathers). Four items were considered irrelevant to parents of 10 to 12 year old children, and were therefore removed from the Norwegian version. These items were: 1) "If this child gets fussy, is giving him/her something to eat or drink the first thing you do?" (from the Emotion regulation subscale), 2) "Do you give this child something to eat or drink if s/he is bored even if you think s/he is not hungry?" (also from the Emotion regulation subscale), 3) "I withhold sweets/desserts from my child in response to bad behavior" (from the Food as reward subscale), and 4) "When he/she says he/she is finished eating, I try to get my child to eat one more (two more, etc.) bites of food" (from the Pressure subscale). This study did not involve development of new items to replace the ones that were removed. Thus, the adapted Norwegian version of the CFPQ consisted of 45 items assumed to tap 12 dimensions of parental feeding practices (dimensions/subscales, items and response formats included in the Norwegian version of the CFPQ are presented in Appendix 1).

#### Related attitude scales

Like Musher-Eizenman & Holub [10], we also asked the parents to respond to items on three related attitude scales adapted from the CFQ [8]: The concern about child overweight scale (3 items), the concern about child underweight scale (3 items) and the responsibility for child eating scale (3 items) (see Appendix 1). These items were included for validation purposes, and they were translated/back translated and pre-tested on parents of 10-to-12-year-olds like the CFPQ items.

### Statistical analyses

SPSS Version 15 was used for the statistical procedures. Prior to psychometric scale analysis, the distribution of scores on each subscale was assessed by calculating mean, standard deviation, skewness and kurtosis values. As suggested by Muthen and Kaplan [29], skewness and kurtosis values lying between -1 and +1 were used as an acceptable range for normality.

Psychometric scale analysis was performed as suggested by Churchill [30]. First, factor analysis (Principal Component Analysis; PCA) was performed on the individual subscales as an initial test of the dimensionality and convergent validity of the scales in our sample. Next, internal consistency for each subscale was assessed by Cronbach's alpha. After that, scale composites were made and bivariate correlations between CFPQ scales were run as an initial test of discriminant validity. According to Churchill [30] and Andersen et al [31], analyses at a subscale level is not always sufficient to reveal all poorly performing items. For that reason, the factor structure and discriminant validity was further tested by running factor analysis (PCA) on the unified 42 item version of the instrument. Finally, like Musher-Eizenman and Holub [10], we ran bivariate correlations between CFPQ subscales and related attitude scales to examine if the scales related to each other in theoretically expected ways (i.e. nomological validity).

For factor analysis, at least three variables per factor is recommended [32]. Consequently, the Emotion regulation and Food as reward subscales were not included in the analyses because they had too few items (one and two items respectively). Thus, the analytical steps described in the previous paragraphs were performed on a 10 subscale, 42 item version of the CFPQ. The suitability of data for factor analysis was assessed by inspection of the correlation matrix, by computing the Kaiser-Meyer-Olkin value (KMO) [33], and by running Bartlett's Test of Sphericity [34] for each subscale as well as for the unified 42 item version of the instrument. Tabachnick and Fidell [35] recommend the presence of coefficients greater than 0.3 in the correlation matrices, KMO values of 0.6 or greater, and significant Bartlett's tests (p < 0.05) for factor analysis to be considered appropriate.

To avoid over- or under-extraction of factors, a combination of the Kaiser criterion (the eigenvalues-greater-than-one-rule) [36], the Monte Carlo PCA for parallel analysis (a simulation method that compares the observed eigenvalues with eigenvalues obtained from a large number of random data sets) [37,38], and substantive evaluation based on previous research, was used for deciding the number of factors to retain. Since there is evidence that some feeding practices are significantly correlated [10], oblique rotation was chosen to clarify the data structure [39,40]. Communalities of 0.5 or higher and/or factor loadings of 0.4 or higher on assigned scale was used as a criterion for convergent validity, while cross loadings of less than 0.4 on any other scale was used as a criterion for discriminant validity [41].

## Results

### Distribution of scores

Mean scores, standard deviations, skewness and kurtosis values of the ten CFPQ subscales and three related attitude scales are presented in Table 1. The skewness and kurtosis values indicated that the scales were relatively normally distributed thus satisfying the normality assumption in multivariate analysis.

**Table 1:** Mean scores, standard deviations, skewness and kurtosis for 10 CFPQ subscales and 3 related attitude scales

	Mean (SD)	Skewness	Kurtosis
*CFPQ subscale (number of items)*			
Child control (5)	2.4 (0.6)	0.49	0.41
Encourage balance and variety (4)	4.5 (0.5)	**-1.04**	0.93
Environment (4)	3.9 (0.7)	-0.43	-0.28
Involvement (3)	3.5 (0.8)	-0.25	-0.47
Modeling (4)	3.9 (0.7)	-0.56	0.31
Monitoring (4)	4.0 (0.6)	-0.50	**1.11**
Pressure (3)	2.8 (1.0)	-0.05	-0.65
Restriction for health (4)	2.9 (1.0)	0.05	-0.78
Restriction for weight control (8)	2.2 (0.8)	0.58	-0.08
Teaching nutrition (3)	4.1 (0.7)	-0.67	-0.10
*Related attitude scales (number of items)*			
Responsibility for child eating (3)	4.0 (0.5)	-0.39	0.62
Concern for child overweight (3)	1.7 (1.0)	**1.32**	0.68
Concern for child underweight (3)	1.8 (1.0)	**1.15**	0.41

*Note: *All response formats are 5-point Likert type scales (see appendix for details). Skewness and kurtosis values exceeding the absolute value of 1 are written in boldfaced type.

### Initial subscale analyses

Initial scale analyses included assessment of each subscale's dimensionality (convergent validity) and internal consistency. Inspection of the subscales' correlation matrices showed consistently significant positive correlations, most of them larger than 0.3. The KMO values for the subscales ranged from 0.54 to 0.87, and Bartlett's Test of Sphericity reached statistical significance (p = 0.000) for all subscales, supporting the factorability of the correlation matrices. PCA with parallel analysis on each individual subscale revealed that 9 out of 10 subscales were unidimensional, whereas one subscale (Environment) showed a two-factor solution as one of its items (item 20: "A variety of healthy foods are available to my child at each meal served at home") loaded onto a second factor. A few very low communality items were also revealed: item10 on the child control subscale (0.24), item 41 on the Modelling subscale (0.24) and item 39 on the Teaching nutrition subscale (0.20). Internal consistency coefficients (Cronbach's alpha) ranged from 0.44 to 0.84 (Table 2).

**Table 2:** Subscale names, item numbers, factor loadings, communalities, internal consistency coefficients (Cronbach's alpha), and variance explained by the first factor (%) for the individual CFPQ subscales

Subscale name (item numbers)	**Factor loadings**, min-max	**Communalities**, min-max	Cronbach's alpha	Variance explained by first factor
Child control (item 5, 6, 8, 9, 10)	0.49-0.66	0.24^b ^-0.44	0.55	37%
Encourage balance and variety (item 11, 22, 24, 35)	0.66-0.78	0.44-0.60	0.66	50%
Environment (item 12, 14, 20, 34)	0.66-0.82 (0.86)^a^	0.60-0.82	0.57	47%
Involvement (item 13, 18, 30)	0.78-0.79	0.61-0,62	0.67	61%
Modeling (item 41, 43, 44, 45)	0.49-0.86	0.24^b ^-0.74	0.66	52%
Monitoring (item 1, 2, 3, 4)	0.74-0.91	0.54-0.82	0.84	70%
Pressure to eat (item 15, 28, 36)	0.57-0.84	0.33-0.71	0.61	57%
Restriction for health (item 19, 26, 37, 40)	0.64-0.80	0.41-0.64	0.73	55%
Restriction for weight (item 16, 25, 27, 31, 32, 33, 38, 42)	0.43-0.80	0.44.0.73	0.83	47%
Teaching nutrition (item 23, 29, 39)	0.45-0.81	0.20^b ^-0.65	0.44	50%

^a ^Item 20 on the Environment subscale did not load onto its assigned scale, but had a high loading onto a second factor.

^b ^The following items had very low communalities: item 10 on the child control subscale, item 41 on the Modeling subscale, and item 39 on the Teaching nutrition subscale.

### Correlations between CFPQ subscales

Discriminant validity was initially assessed by running bivariate correlation analysis between the CFPQ subscales. Before running correlation analysis, composites were made by averaging the item scores on each subscale. Since there is no reason to believe that the items are of different importance [10], all items were weighted equally. Discriminant validity of the CFPQ subscales was supported, as the majority of correlations between scales were weak to moderate (0.01-0.56) (Table 3). The highest correlations were found between the Restriction for health and Restriction for weight control subscales (r = 0.56, p < 0.01), and between the Teaching nutrition and Encourage balance and variety subscales (r = 0.52, p < 0.01). However, these correlations were not large enough to compromise the discriminant validity of the scales (see discussion).

**Table 3:** Bivariate correlations between the 10 CFPQ subscales

	CC	Enc	Env	Inv	Mod	Mon	Pre	RH	RW	Teach
Child control (CC)	-									
Encourage bal./var. (Enc)	**-.24**	**-**								
Environment (Env)	**-.18**	**.26**	**-**							
Involvement (Inv)	.04	**.31**	**.16**	**-**						
Modeling (Mod)	**-.14**	**.43**	**.27**	**.21**	**-**					
Monitoring (Mon)	**-.22**	**.20**	**.16**	.04	**.11**	**-**				
Pressure to eat (Pre)	-.02	-.03	**-.16**	-.05	.06	-.08	**-**			
Restriction for health (RH)	.03	.08	-.06	-.02	**.16**	-.04	-.01	**-**		
Restriction for weight (RW)	-.03	.09	.05	.08	**.17**	.01	**-.12**	**.56**	**-**	
Teaching nutrition (Teach)	**-.19**	**.52**	**.34**	**.28**	**-.10**	**.13**	**-.10**	.02	**.11**	**-**

*Note: *Correlations in bold are significant at the .01 level.

### Analysis of the unified 42 item version of the CFPQ

Since analysis at a subscale level is not always sufficient to reveal all poorly performing items, factor structure and discriminant validity was further assessed by running factor analysis (PCA) on the unified 42 item version of the instrument. Inspection of the correlation matrix for the complete 42 item version revealed (as expected) the presence of many correlation coefficients of 0.3 and above. The KMO value was 0.82, and Bartlett's Test of Sphericity showed statistical significance (p = 0.000), supporting the factorability of the correlation matrix. The Kaiser criterion (which tends to over-extract factors) suggested that 10 factors should be retained, while parallel analysis (which is one of the most recommendable rules for factor-extraction) suggested 8 factors. Based on these results, we compared 8-, 9-, 10- and 11-factor solutions to decide how many factors to retain. In our sample, the 10-factor solution was found to be conceptually more reasonable than the others. In this solution the majority of items clustered to form factors corresponding with the original instrument, showing a simple structure, and explaining 57% of the variance in our data (Table 4). However, there were some differences worth noting: the items on the Encourage balance and variety and the Teaching nutrition subscales loaded onto the same factor. In addition, the four items on the Environment subscale split into two different factors, one containing items reflecting availability of healthy foods in the home environment, and another one containing items reflecting availability of unhealthy foods in the home environment.

**Table 4:** Factor structure of the unified 42 item version of the CFPQ (our 10-factor solution), and variance explained for each factor

CFPQ items	CC	**Enc/Teach**^**b**^	**Env_U**^**c**^	**Env_H**^**c**^	Inv	Mod	Mon	Pre	RH	RW
CC5	0.73									
CC9	0.62									
CC6	0.61									
CC8	0.51									
CC10^a^										
Enc22		0.69								
Enc35		0.61								
Enc24		0.53								
Enc11		0.48								
Env14			0.89							
Env34			0.85							
Env20				0.64						
Env12				0.57						
Inv13					0.79					
Inv18					0.76					
Inv30					0.74					
Mod44						0.87				
Mod43						0.78				
Mod45						0.65				
Mod41				0.57^d^						
Mon2							0.92			
Mon1							0.91			
Mon4							0.81			
Mon3							0.71			
Pre36								0.77		
Pre28								0.73		
Pre15								0.64		
RH40									0.65	
RH26									0.62	
RH19									0.61	
RH37									0.50	
RW25										0.82
RW31										0.80
RW27										0.75
RW33										0.64
RW32										0.61
RW38										0.60
RW16										0.50
RW42 Teach23		0.64		0.40^e^						
Teach29 Teach39		0.40 0.42								
Variance expl. (%)	2.5	11.0	2.9	4.1	4.7	3.0	6.8	5.1	3.7	13.1

*Note: *Original CFPQ subscales (and item prefixes) are labeled as follows: Child control (CC), Encourage balance and variety (Enc), Environment (Env), Involvement (Inv), Modeling (Mod), Monitoring (Mon), Pressure (Pre), Restriction for health (RH), Restriction for weight (RW), Teaching nutrition (Teach). Only factor loadings higher than the absolute value of 0.40 are reported.

^a ^Item 10 on the original Child control subscale did not have a substantial loading onto any factors in our solution.

^b ^Items from the Encourage balance and variety and Teaching nutrition subscales loaded onto the same factor, creating a new Enc/Teach factor.

^c ^The original Environment subscale was not confirmed, but was split into two different factors reflecting availability of healthy foods in the home environment (Env_H) and availability of unhealthy foods in the home environment (Env_U) respectively.

^d ^Item 41 from the Modeling subscale did not load onto the Modeling factor, but onto the new Env_H factor.

^e ^Item 42 from the Restriction for weight (RW) subscale did not load onto the RW factor, but onto the new Env_H factor.

Also important to note, is that item 10 on the Child control subscale ("Do you allow this child to leave the table when s/he is full, even if your family is not done eating?") did not have a substantial loading onto any factor in our solution. Furthermore, one item on the Modeling subscale (item 41: "I model healthy eating for my child by eating healthy myself"), and one item on the Restriction for weight subscale (item 42: "I often put my child on a diet to control his/her weight"), did not load onto their assigned scales, but loaded together with the items reflecting availability of healthy foods in the home environment (see discussion).

### Correlations between CFPQ subscales and related attitude scales

Nomological validity was assessed by running bivariate correlation analysis between the CFPQ subscales in our 10-factor solution and related attitude scales derived from Birch et al [8] (see Appendix 1). Theoretically expected relations between CFPQ subscales and related attitude scales were supported by our analyses (see Table 5), thus placing the CFPQ subscales in the nomological network of the multidimensional domaine of parental feeding behavior (see discussion).

**Table 5:** Bivariate correlations between CFPQ subscales (our 10-factor solution) and related attitude scales

	CC	Enc/Teach	Env_U	Env_H	Inv	Mod	Mon	Pre	RH	RW
Responsibility for child eating	**-.16**	.04	.08	**.24**	**.09**	**.36**	**.20**	.03	**.20**	**.33**
Concern overweight	.04	.03	-.03	.01	-.01	**.10**	**-.10**	**-.14**	**.47**	**.64**
Concern underweight	**.14**	.07	**-.14**	-.00	.04	.03	**-.16**	**.36**	.06	-.06

*Note: *Correlations in bold are significant at the .01 level.

To sum up, the results from our quite comprehensive scale analyses largely supported the validity and internal consistency reliability of the CFPQ subscales in the present sample. However, a few problems were revealed, and these problems form the basis of the discussion below.

## Discussion

The aim of the present study was to test the validity of a slightly adapted version of the CFPQ with Norwegian parents of 10 to12 year old children. Analyses of both the individual subscales and a unified 42 item version of the instrument suggested reasonable validity of the CFPQ in our sample.

The initial scale analyses included assessment of subscale dimensionality (convergent validity) and internal consistency. Reasonable convergent validity and internal consistency was found for most scales. However, there were indications of some problems within the following four subscales: Child control, Environment, Modelling, and Teaching nutrition. We found some very low communality items within the Child control, Modelling and Teaching nutrition subscales, and the Environment subscale showed a two-factor solution, thus indicating some problems with the convergent validity of these scales. Moreover, the low alphas found in three of these scales may be questioned (Child control = 0.55, Teaching nutrition = 0.44, Environment = 0.57). Some low alphas were also found by Musher-Eizenman & Holub (2007) (e.g. Encourage balance and variety = 0.58 for American mothers) and Musher-Eizenman et al [15] (e.g. Teaching nutrition = 0.54 and 0.56 for French mothers and fathers respectively). However, it is important to note that all CFPQ subscales have few items. According to Cortina [42], it is well known that the number of items has an effect on alpha, especially at low levels of average item inter-correlation. That is, if a scale has enough items (e.g. more than 20), then it can have an alpha of ≥ 0.70 even when the correlation among items are very small [42]. Thus, lower values of alpha can be expected from shorter scales like the subscales of the CFPQ. Developing survey instruments always involves a trade-off between internal consistency (using multiple items) and practicality. The CFPQ is an instrument aiming to tap many different aspects of feeding practices. Using only a few items in each subscale makes it less tiresome, and therefore more applicable. However, one may question if the brief subscales of the CFPQ sufficiently captures the different aspects of feeding practices.

Initial testing of discriminant validity by running correlation analyses between the CFPQ subscales revealed some substantial correlations, but these were not large enough to compromise the discriminant validity of the scales [40]. The correlation (r = 0.56, p < 0.01) between the Restriction for weight control and Restriction for health subscales could be expected, as these scales represent conceptually close constructs. Musher-Eizenman and Holub [10] indicated that parents may not spontaneously differentiate between restriction motivated by weight and by health, suggesting that parents who limit or restrict child food intake for weight control reasons may also be doing so for health reasons (or vice versa). Yet, Musher-Eizenman and Holub [43] was the first to articulate the distinction between restriction for health reasons and restriction for weight control reasons. They argue in favor of the distinction between different types of restrictive feeding, as there may be many different motivations behind the restriction, including child health outcomes, child weight loss or maintenance, to teach the child healthy eating habits for the future, or for religious or ethical beliefs. In their directions for future research, they suggest further exploration of the effect of different restrictive feeding practices on child eating, weight and health outcomes. The correlation (r = 0.52, p < 0.01) between the Encourage balance and variety and Teaching nutrition subscales was also expected, as these scales both deal with explicit nutrition communication with the child. The relation and discrimination between these scales are further discussed below.

When running factor analysis on the unified 42-item version of the CFPQ, a 10-factor solution, largely corresponding with the original instrument developed by Musher-Eizenman and Holub [10], was found to be conceptually sound in our sample. However, there were some small, but noteworthy differences in factor structure between our solution and the one suggested by Musher-Eizenman and Holub [10]: In our solution the items on the Encourage balance and variety and Teaching nutrition subscales clustered together to form one factor. Moreover, the four items on the Environment subscale split into two different factors reflecting availability of healthy and unhealthy foods respectively. Also worth noting, is that the following items did not load onto their assigned scales: item 10 on the Child control subscale, item 41 on the Modeling subscale, and item 42 on the Restriction for weight subscale.

If we take a closer look at the problematic scales and items revealed by our analyses, it may seem as if some of the items are not conceptualized in an adequate way. Starting with the low-communality item 10 ("Do you allow this child to leave the table when s/he is full, even if your family is not done eating?") on the Child control subscale, this item might reflect a breach of meal-related social norms rather than child control over what and when to eat. In other words, leaving the table when full before the rest of the family is done eating, might reflect a breach of good table manners, an ideal learned through family meals in most Western cultures [44]. Thus, item 10 did not seem feasible as a measure of child control over food intake in our sample.

Moving on to the Environment subscale, the items of this scale split into two different factors with items 12 ("Most of the food I keep in the house is healthy") and 20 ("A variety of healthy foods are available to my child at each meal served at home") loading onto one factor reflecting to what extent healthy foods are available in the home environment, and items 14 ("I keep a lot of snack food in my house") and 34 ("I keep a lot of sweets in my house") loading onto a second factor reflecting to what extent unhealthy foods are available in the home environment. Both correlation analysis between the two "new" factors (r = 0.21, p < 0.01) and face validity supports this distinction. Thus, the items on the original Environment subscale seem to specify two different kinds of behavior (having healthy foods available in the home vs. having unhealthy foods available in the home) in our sample.

Initial analysis of the Modelling subscale revealed one low-communality item that needed further investigation (item 41 "I model healthy eating for my child by eating healthy myself"). This item is distinct from the other items on the Modelling scale, as it seems to reflect a general, "passive" form of modelling (...eating healthy myself...), while the remaining three items involve specific, "active" components (...eating healthy foods *in front of my child*..., ...*show enthusiasm *about eating healthy foods..., ...*show how much I enjoy *eating healthy foods...). This distinction was supported when running factor analysis on the unified 42 item CFPQ; item 41 did not load onto the Modelling factor, but onto the factor reflecting availability of healthy foods in the home environment. One obvious assumption for healthy eating is availability of healthy foods. Thus, if parents practice healthy eating, healthy foods are most likely available in the home. Collectively, studies do suggest that readily available and easily accessible healthy foods within the home are likely to enhance healthy eating among families [45]. To sum up on this; healthy eating practices among parents might be more related to the availability of healthy foods in the home environment than to "active" modeling of healthy eating.

The initial scale analyses revealed that the Teaching nutrition subscale had one very low communality item (item 39 "I tell my child what to eat and what not to eat without explanation") and two high communality items (item 23 "I discuss with my child why it's important to eat healthy foods", and item 29 "I discuss with my child the nutritional value of foods"). Item 39 seems to reflect an authoritarian interaction with the child, while a more democratic, authoritative mode of interaction is reflected by items 23 and 29. In the feeding domain, authoritarian practices include parental control and indisputable instructions on what to eat [46,47], while authoritative practices include using discussion, negotiations, and reasoning for desirable eating behavior [48]. In light of this, item 39 seems to reflect a different type of parental food-related behavior than items 23 and 29, which might explain its lack of communality with the latter two. A suboptimal performance of the Teaching about nutrition scale was also found by Musher-Eizenman and co-workers [15].

When running factor analysis on the unified 42 item version of the CFPQ the items on the Teaching nutrition and Encourage balance and variety subscales loaded highly onto the same factor, indicating a lack of discriminant validity between the scales. A certain overlap between these measures could be expected since they both deal with explicit nutrition communication with the child. Although the bivariate correlation between them (r = 0.52, p < 0.01) was not high enough to suggest a complete conceptual overlap, factor analysis did not support the discriminant validity of the two scales in our sample.

While item 42 ("I often put my child on a diet to control his/her weight") on the Restriction for weight subscale performed well in the initial analysis on the individual subscales, factor analysis of the unified version of the CFPQ showed that item 42 did not load onto its assigned scale, but loaded (together with item 41) onto the factor reflecting availability of healthy foods in the home environment. Like for healthy eating, (successful) dieting also requires availability of healthy foods in the home. Thus, item 42 might be more related to the availability of healthy foods in the home environment than to restriction for weight control reasons. If we see this in light of the general recommendation about not focusing on dieting in front of children and adolescents [49], the unsubstantial loading of item 42 on its assigned scale was not totally unexpected. Furthermore, the CFPQ was first developed and tested in the US [10], where child overweight and obesity is substantially more prevalent than in Norway [50,51]. Thus, one might speculate if this item is more appropriate in an American than in a Norwegian setting.

Regarding nomological validity, significant correlations between CFPQ subscales in our 10-factor solution and related attitude scales supported theoretically expected relations. For instance; parents who were concerned about their child being or becoming overweight reported more restrictive feeding practices of both types, whereas parents who were concerned about their child being or becoming underweight reported more pressure to eat. Furthermore, parents feeling responsible for child eating reported less child control over feeding interactions, a healthier home environment, more modelling, monitoring, encouragement and teaching about nutrition, and more restriction of both types.

To sum up: our findings largely supported the validity of a slightly adapted, 42 item version of the CFPQ with parents of 10 to12 year old children in a Norwegian setting. Although some subscales and items seemed problematic as a result of our statistical scale analyses, face validity indicated that most of these items still were relevant for measuring feeding practices in parents of 10-to-12-year-olds. Furthermore, it is important to note that our findings are sample specific, and thus cannot be used as a sole foundation for changing the original CFPQ subscales. The CFPQ has previously been validated with parents of younger children, and in other cultural settings (USA; Musher-Eizenman & Holub, 2007, and France; Musher-Eizenman et al., 2009). Thus, some differences when it comes to factor structure and other validity measures between these studies and the present one are not unexpected.

### Strengths and limitations

Among the strengths of this study is its large sample size. According to Guadagnoli and Velicer [52], a sufficiently large sample size is one of the most important factors for determining a stable factor structure. Pett, Lackey and Sullivan [40] recommend that there be at least 10 to 15 subjects per item, preferably aiming for a sample size of 500 or more. We more than satisfy these recommendations with our sample of 963 respondents. Furthermore, most previous validation studies on feeding practices measures have focused on parents of young children and on rather parsimonious instruments largely tapping aspects of parental control over child eating behavior. Thus, the present study extends the current literature by validating a multi-dimensional feeding practices instrument with parents of older children. We believe this is a relevant contribution, as valid instruments are needed to assess a wider range of feeding practices in diverse groups of parents, including parents of older children and adolescents [6].

A few limitations of this study need comments. The findings are limited to Norwegian parents of pre-adolescent children. Furthermore, four items were excluded from the Norwegian version of the CFPQ, and only 10 out of 12 subscales were validated in the present study (thus, a reduced version of the CFPQ was tested). Second, the study sample was a cluster sample drawn from a confined geographic area (two municipalities in the South-Western part of Norway). However, as Norway is a rather homogeneous country [53], we believe the results are likely to be generalized to other areas in Norway.

## Conclusions

The psychometric properties of the slightly adapted Norwegian version of the CFPQ were found to be surprisingly similar to those of the original CFPQ. Thus, we suggest that the CFPQ, with some modifications, is a valid tool for assessing parental feeding practices with parents of 10-to-12-year-olds in a Norwegian setting. The good response rate (66%) indicates that the content of the CFPQ is considered relevant by this group of the population. However, the CFPQ is not yet an established instrument, and the present study can be considered part of an early phase validation process. Although our validation of a Norwegian version of the CFPQ with parents of 10-to-12-year olds yielded positive results for most subscales and items, we suggest further fine-tuning of the instrument and inclusion of new items to make it an even more complete instrument for use with parents of older children and adolescents. Future fine-tuning and item generation should involve further exploration of the different dimensions of feeding practices, and the weights given to the different dimensions, through qualitative research in the target population. Based on our results, special attention should be given to the dimensions of restrictive feeding practices and the dimensions reflecting home food environment and nutrition communication between parents and children. An expanded cross-cultural adaptation and further improvement of the psychometric quality of this instrument becomes even more important if there is an interest in comparing results from research conducted in different cultures and settings. Nevertheless, our results indicate that the CFPQ is a promising tool for future comparative studies and much needed accumulation of knowledge about parent-child feeding interactions.

## Competing interests

The authors declare that they have no competing interests.

## Authors' contributions

ELM designed the study, collected and analyzed the data, and drafted the manuscript. NCØ and TØ supervised the study and contributed to the analyses and writing of the article. All of the authors read and approved the final manuscript.

## Appendix 1

Includes subscale names, brief operational definition of subscales, and items retained in the Norwegian version of the CFPQ and the related attitude scales adapted from the CFQ. Item numbers indicate the order in which they were presented in the survey questionnaire. Items numbered 1-11 utilize a 5-point "frequency scale"; never, rarely, sometimes, mostly, always. Items numbered 12-48 utilize a 5-point "agreement scale"; disagree, slightly disagree, neutral, slightly agree, agree. Items numbered 49-54 utilize a 5-point "concern scale"; unconcerned, a little concerned, concerned, fairly concerned, very concerned. Items marked with an **R **were reversed coded.

### CFPQ subscales and items

**Child control **- parents allow the child control of his/her eating behaviors and parent-child feeding interactions

5. Do you let your child eat whatever s/he wants?

6. At dinner, do you let this child choose the foods s/he wants from what is served?

8. If this child does not like what is being served, do you make something else?

9. Do you allow this child to eat snacks whenever s/he wants?

10. Do you allow this child to leave the table when s/he is full, even if your family is not done eating?

**Emotion regulation **- parents use food to regulate the child's emotional status

7. Do you give this child something to eat or drink if s/he is upset even if you think s/he is not hungry?

**Encourage balance and variety **- parents promote well-balanced food intake, including the consumption of varied foods and healthy food choices

11. Do you encourage this child to eat healthy foods before unhealthy ones?

22. I encourage my child to try new foods

24. I tell my child that healthy foods taste good

35. I encourage my child to eat a variety of foods

**Environment **- parents make (un)healthy foods available in the home

12. Most of the food I keep in the house is healthy

14. I keep a lot of snack food (potato chips, Doritos, cheese puffs) in my house **R**

20. A variety of healthy foods are available to my child at each meal served at home

34. I keep a lot of sweets (candy, ice cream, cake, pastries) in my house **R**

**Food as reward **- parents use food as reward for child behavior

17. I offer my child his/her favorite foods in exchange for good behavior

21. I offer sweets (candy, ice cream, cake, pastries) to my child as a reward for good behavior

**Involvement **- parents encourage child's involvement in meal planning and preparation

13. I involve my child in planning family meals

18. I allow my child to help prepare family meals

30. I encourage my child to participate in grocery shopping

**Modeling **- parents actively demonstrate healthy eating for the child

41. I model healthy eating for my child by eating healthy myself

43. I try to eat healthy foods in front of my child, even if they are not my favorite

44. I try to show enthusiasm about eating healthy foods

45. I show my child how much I enjoy eating healthy foods

**Monitoring **- parents keep track of child's intake of less healthy foods

1. How much do you keep track of the sweets (candy, ice cream, cake, pastries) that you child eats?

2. How much do you keep track of the snack food (potato chips, Doritos, cheese puffs) that your child eats?

3. How much do you keep track of the high-fat foods that your child eats?

4. How much do you keep track of the sugary drinks this child drinks?

**Pressure **- parents pressure the child to consume more foods at meals

15. My child should always eat all of the food on his/her plate

28. If my child says, "I'm not hungry", I try to get him/her to eat anyway

36. If my child eats only a small helping, I try to get him/her to eat more

**Restriction for health **- parents control the child's food intake with the purpose of limiting less healthy foods and sweets

19. If I did not guide or regulate my child's eating, s/he would eat too much of his/her favorite foods

26. If I did not guide or regulate my child's eating, s/he would eat too many junk foods

37. I have to be sure that my child does not eat too much of his/her favorite foods

40. I have to be sure that my child does not eat too many sweets (candy, ice cream, cake, pastries)

**Restriction for weight control **- parents control the child's food intake with the purpose of decreasing or maintaining the child's weight

16. I have to be sure that my child does not eat too many high-fat foods

25. I encourage my child to eat less so s/he won't get fat

27. I give my child small helpings at meals to control his/her weight

31. If my child eats more than usual at one meal, I try to restrict his/her eating at the next meal

32. I restrict the food my child eats that might make him/her fat

33. There are certain foods my child shouldn't eat because they will make him/her fat

38. I don't allow my child to eat between meals because I don't want him/her to get fat

42. I often put my child on a diet to control his/her weight

**Teaching about nutrition **- parents use explicit didactic techniques to encourage the consumption of healthy foods

23. I discuss with my child why it's important to eat healthy foods

29. I discuss with my child the nutritional value of foods

39. I tell my child what to eat and what not to eat without explanation **R**

### Related attitude scales and items adapted from the CFQ

**Responsibility for child eating **- parents feel responsible for their child's eating

46. I feel that I have an important role in establishing lifelong eating habits in my child

47. I feel responsible for determining portion sizes for my child

48. I feel responsible for providing a healthy diet for my child

**Concern for child overweight **- parents are concerned about their child being/becoming overweight

49. How concerned are you about your child eating too much when you are not around him/her?

50. How concerned are you about your child having to diet to maintain a desirable weight?

51. How concerned are you about your child becoming overweight?

**Concern for child underweight **- parents are concerned about their child being/becoming underweight

52. How concerned are you about your child eating too little when you are not around him/her?

53. How concerned are you about your child having to eat more to maintain a desirable weight?

54. How concerned are you about your child becoming underweight?

## Pre-publication history

The pre-publication history for this paper can be accessed here:

http://www.biomedcentral.com/1471-2288/11/113/prepub

## References

[B1] Nicklas T Baranowski T Baranowski J Cullen K Rittenberry L Olvera N Family and Child-care Provider Influences on Preschool Children's Fruit, Juice and Vegetable Consumption Nutr Rev 2001 59 7 10.1111/j.1753-4887.2001.tb07014.x 11475448

[B2] Savage JS Fisher JO Birch LL Parental influence on eating behaviour: conception to adolescence J Law Med Ethics 2007 35 22 34 10.1111/j.1748-720X.2007.00111.x 17341215 PMC2531152

[B3] Golan M Crow S Targeting Parents Exclusively in the Treatment of Childhood Obesity: Long-Term Results Obes Res 2004 12 357 361 10.1038/oby.2004.45 14981230

[B4] Hart KH Bishop JA Truby H Changing children's diets: developing methods and messages J Hum Nutr Diet 2003 16 365 370

[B5] Zeinstra GG Cognitive development and children's perceptions of fruit and vegetables; a qualitative study Int J Behav Nutr Phys Act 2007 4 30 10.1186/1479-5868-4-30 PMC1941844 17620131

[B6] Faith MS Storey M Kral TVE Pietrobelli A The Feeding Demands Questionnaire: Assessment of parental demand cognitions concerning parent-child feeding relations J Am Diet Assoc 2008 108 624 630 10.1016/j.jada.2008.01.007 18375218 PMC2917044

[B7] Faith MS Scanlon KS Birch LL Francis LA Sherry B Parent-Child Feeding Strategies and Their Relationships to Child Eating and Weight Status Obes Res 2004 12 1711 1722 10.1038/oby.2004.212 15601964

[B8] Birch LL Fisher JO Grimm-Thomas K Markey CN Sawyer R Johnson SL Confirmatory factor analyses of the Child Feeding Questionnaire: a measure of parental attitudes, beliefs and practices about child feeding and obesity proneness Appetite 2001 36 201 210 10.1006/appe.2001.0398 11358344

[B9] Birch LL Fisher JO Mothers' child feeding practices influence daugthers' eating and weight Am J Clin Nutr 2000 71 1054 1061 10799366 10.1093/ajcn/71.5.1054 PMC2530928

[B10] Musher-Eizenman DR Holub SC Comprehensive Feeding Practices Questionnaire: Validation of a New Measure of Parental Feeding Practices J Pediatr Psychol 2007 1 13 10.1093/jpepsy/jsm037 17535817

[B11] Hendy HM Raudenbush B Effectiveness of teacher modeling to encourage food acceptance in preschool children Appetite 2000 34 61 76 10.1006/appe.1999.0286 10744893

[B12] Lee Y Birch LL Diet quality, nutrient intake, weight status and feeding environments of girls meeting or exceeding the American Academy of Pediatrics recommendations for total dietary fat Minerva Pediatr 2002 54 179 186 12070476 PMC2533131

[B13] Wardle J Cooke L Gibson L Sapochnik M Sheiham A Lawson M Increasing children's acceptance of vegetables; a randomized trial of parent-led exposure Appetite 2003 40 155 162 10.1016/S0195-6663(02)00135-6 12781165

[B14] Blanchette L Brug J Determinants of fruit and vegetable consumption among 6-12-year-old children and effective interventions to increase consumption J Hum Nutr Dietet 2005 18 431 443 10.1111/j.1365-277X.2005.00648.x 16351702

[B15] Musher-Eizenman DR de Lauzon-Guillain B Holub SC Leporc E Charles M Child and parent characteristics related to parental feeding practices. A cross-cultural examination in the US and France Appetite 2009 52 89 95 10.1016/j.appet.2008.08.007 18789986 PMC3808174

[B16] Kaur H Li C Nazir N Choi WS Resnicow K Birch LL Ahluwalia JS Confirmatory factor analysis of the child-feeding questionnaire among parents of adolescents Appetite 2006 47 36 45 10.1016/j.appet.2006.01.020 16624444

[B17] Hanson NI Neumark-Sztainer D Eisenberg ME Story M Wall M Associations between parental report of the food home environment and adolescent intakes of fruits, vegetables and dairy foods Public Health Nutr 2005 8 77 85 10.1079/PHN2005661 15705248

[B18] Boutelle K Lytle L Murray D Birnbaum A Story M Perception of the family mealtime environment and adolescent mealtime behaviour: Do adults and adolescents agree? J Nutr Educ 2001 33 128 133 10.1016/S1499-4046(06)60181-4 11953227

[B19] Koivisto U Sjøden P Reasons for rejection of food items in Sweedish families with children aged 2-17 Appetite 1996 26 89 103 10.1006/appe.1996.0007 8660035

[B20] Erikson EH Youth: Change and challenge 1963 New York: Basic books

[B21] Shepherd R Dennison C Influences of adolescent food choice Proc Nutr Soc 1996 55 345 357 10.1079/PNS19960034 8832805

[B22] Robinson S Children's perceptions of who controls their foods J Hum Nutr Dietet 2000 13 163 171 10.1046/j.1365-277x.2000.00229.x 12383123

[B23] Cavadini C Decarli B Dirren H Cauderay M Narring F Michaud P Assessment of adolescent food habits in Switzerland Appetite 1999 32 97 106 10.1006/appe.1998.0202 9989920

[B24] Story M Neumark-Sztainer D French S Individual and environmental influences on adolescent eating behaviours J Am Diet Assoc 2002 102 40 51 10.1016/S0002-8223(02)90421-9 11902388

[B25] Lytle L Nutrition education for school aged children J Nutr Educ 1995 27 298 311

[B26] Bissonette M Contento I Adolescents' perspectives and food choice behaviours in terms of the environmental impacts of food production practices: application of a psycosocial model J Nutr Educ 2001 33 72 82 10.1016/S1499-4046(06)60170-X 12031187

[B27] Hart KH Bishop JA Truby H An investigation into school children's knowledge and awareness of food and nutrition J Hum Nutr Dietet 2002 15 129 140 10.1046/j.1365-277X.2002.00343.x 11972742

[B28] Lov om medisinsk og helsefaglig forskning (Helseforskningsloven) 2008 Norway: Helse- og omsorgsdepartementet

[B29] Muthen B Kaplan D A comparison of some methodologies for the factor analysis of non-normal Likert variables Brit J Math Stat Psy 1985 38 171 189 10.1111/j.2044-8317.1985.tb00832.x

[B30] Churchill GA A paradigm for developing better measures of marketing constructs J Marketing Res 1979 16 64 73 10.2307/3150876

[B31] Anderson CB Hughes SO Fisher JO Nicklas TA Cross-cultural equivalence of feeding beliefs and practices: The psychometric properties of the child feeding questionnaire among Blacks and Hispanics Prev Med 2005 41 521 531 10.1016/j.ypmed.2005.01.003 15917048

[B32] Kim J-O Mueller CW Factor analysis: Statistical methods and practical issues 1978 Thousand Oaks, CA: Sage Publications

[B33] Kaiser H An index of factorial simplicity Psychometrica 1974 39 31 36 10.1007/BF02291575

[B34] Bartlett MS A note on the multiplying factors for various chi square approximations J R Stat Soc 1954 16 296 298

[B35] Tabachnick BG Fidell LS Using Multivariate Statistics 2007 5 Boston: Pearson Education

[B36] Kaiser H The application of electronic computers to factor analysis Educ Psychol Meas 1960 20 141 151 10.1177/001316446002000116

[B37] Horn JL A rationale and test for the number of factors in factor analysis Psychometrika 1965 30 179 185 10.1007/BF02289447 14306381

[B38] Watkins MW MonteCarlo PCA for parallel analysis 2000 State College, PA: Ed and Psych Associates computer software

[B39] Costello AB Osborne JW Best practices in exploratory factor analysis: four recommendations for getting the most from your analysis Practical Assess Res Eval 2005 10 7

[B40] Pett MA Lackey NR Sullivan JJ Making Sense of Factor Analysis. The Use of Factor Analysis for Instrument Development in Health Care Research 2003 Thousand Oaks, California: Sage Publications

[B41] Hair JF Black WC Babin BJ Anderson RE Multivariate Data Analysis. A Global Perspective 2010 7 Upper Saddle River, New Jersey: Pearson Education, Inc.

[B42] Cortina JM What is coefficient alpha? An examination of theory and applications J Appl Psychol 1993 78 98 104

[B43] Musher-Eizenman DR Holub SC Flamenbaum RK Children's eating in the absence of hunger: The role of restrictive feeding practices Childhood Obesity and Health Research 2006 New York: Nova Science Publishers, Inc. 135 156

[B44] Larson RW Branscomb KR Wiley AR Forms and functions of family mealtimes: Multidisciplinary perspectives New Dir Child Dev 2006 11 1 15 10.1002/cd.152 16646496

[B45] Story M Kaphingst KM Robinson-O'Brien R Glanz K Creating healthy food and eating environments:policy and environmental approaches Annu Rev Public Health 2008 29 253 272 10.1146/annurev.publhealth.29.020907.090926 18031223

[B46] Birch LL McPhee L Shoba BC Steinberg L Krehbiel R Clean up your plate: Effects of child feeding practices on the conditioning of meal size Learn Motiv 1987 18 301 317 10.1016/0023-9690(87)90017-8

[B47] Klesges RC Stein RJ Eck LH Isbell TR Klesges LM Parental influence on food selection in young children and its relationships to childhood obesity Am J Clin Nutr 1991 55 859 864 10.1093/ajcn/53.4.859 2008864

[B48] Ianotti RJ O'Brien RW Spillman DM Parental and peer influences on food consumption of preschool African-American children Per Mot Skills 1994 79 747 752 10.2466/pms.1994.79.2.747 7870497

[B49] Rosenvinge JH Børresen R Kan man forebygge spiseforstyrrelser? Tidsskr Nor Legeforen 2004 15 1943 1946 15306865

[B50] Phipps SA Burton PS Osberg LS Lethbridge LN Poverty and the extent of child obesity in Canada, Norway and the United States Obes Rev 2006 7 5 12 10.1111/j.1467-789X.2006.00217.x 16436098

[B51] Janssen I Katzmarzyk PT Boyce WF Vereecken C Mulvihill C Roberts C Currie C Pickett W Comparison of overweight and obesity prevalence in school-aged youth from 34 countries and their relationships with physical activity and dietary patterns Obes Rev 2005 6 123 132 10.1111/j.1467-789X.2005.00176.x 15836463

[B52] Guadagnoli E Velicer WF Relation of sample size to the stability of component patterns Psychol Bull 1988 103 265 275 3363047 10.1037/0033-2909.103.2.265

[B53] Bere E Klepp K-I Changes in accessibility and preferences predict children's future fruit and vegetable intake Int J Behav Nutr Phys Act 2005 2 15 1 8 16216124 10.1186/1479-5868-2-15 PMC1262749

